# Gastrodermis ultrastructure of the different life stages of the polyopisthocotylean monogenean gill parasite *Discocotyle sagittata*

**DOI:** 10.1007/s00436-021-07286-6

**Published:** 2021-08-18

**Authors:** Joanne Cable, Mohamed Mohamed El-Naggar

**Affiliations:** 1 School of Biosciences, Cardiff University, Cardiff, CF10 3AX UK; 2 Zoology Department, Faculty of Science, Mansoura University, Mansoura, Egypt

**Keywords:** Ultrastructure, Gastrodermis, Monogenea, Polyopisthocotylea

## Abstract

The polyopisthocotylean *Discocotyle sagittata* is a blood-feeding monogenean that infects the gill lamellae of rainbow trout, *Oncorhynchus mykiss*, and brown trout, *Salmo trutta*. The ultrastructure of their alimentary tract, at different stages of the life cycle, was previously unknown. Here, we show that the gastrodermis of the oncomiracidium, subadult, and adult *D. sagittata* follows the same structural organization as that of other blood-feeding polyopisthocotyleans, being composed of digestive cells alternating with a connecting syncytium. Digestive cells of the oncomiracidium are found in three developmental forms: undifferentiated, developing differentiated, and differentiated (presumably functioning) cells whereas those of adult and subadult are present in a single functioning state with variable size and content. The apical cytoplasm of adult digestive cells forms conical outgrowths, a feature which is absent in the oncomiracidium. The connecting syncytium of the oncomiracidium has no evidence of metabolic activity, while that of adult and subadult is metabolically active. The lamellae of the connecting syncytium of adults and subadults are more numerous and larger, and their terminal portions are expanded, compared with those of the oncomiracidium. Parallel, tubular, membranous structures are characteristic of the apical cytoplasm of the connecting syncytium of the oncomiracidium. Luminal lamella in the oncomiracidium, subadult, and adult form balloon-like structures enclosing some luminal contents, but those of the oncomiracidium are larger, bounded by nucleated cytoplasmic layer, and enclose more luminal contents. The possible functions of these structures and mechanism of digestion in both oncomiracidium and adult are discussed.

## Introduction

Monogenean ectoparasites typically browse for nutrients from the surface of their fish hosts. The monopisthocotylean monogeneans typically feed on epidermal cells and mucus (Kearn 1963), and their gastrodermis consists of a monotypic layer of digestive cells (Halton and Jennings 1965; Halton and Stranock 1976; Kearn 1979; Kritsky et al. 1994; Cable et al. 1997, 2002; Arafa et al. 2013). In contrast, most polyopisthocotyleans feed on blood (Llewellyn 1954; Kearn 1963; Halton and Jennings 1965) with a gastrodermis consisting of digestive cells and connecting syncytium, which forms a sheet-like lining over the cecal wall and is perforated at intervals by the apical regions of the digestive (hematin) cells (Halton et al. 1968; Rohde 1973; Poddubnaya et al. 2015). There are exceptions though with blood pigments observed in a few monopisthocotyleans (Kearn 1963; Fournier 1978; Buchmann et al. 1987) and the polystomatids *Polystomoides*, *Polystomoidella*, *Neopolystoma* (see Allen and Tinsley 1989), *Apalonotrema*, *Aussietrema*, and *Fornixtrema* du Preez and Verneau 2020 and *Oculotrema* Stunkard 1924 feed on epithelial cells and mucus, the diet typical of monopisthocotyleans.

The polyopisthocotylean *Discocotyle sagittata* (Leukart 1842) Diesing 1850 is a blood-feeding monogenean that infects the secondary gill lamellae of salmonids (rainbow trout, *Oncorhynchus mykiss* Walbaum 1792 and brown trout, *Salmo trutta* Linnaeus 1758). Although these worms have been the subject of taxonomical, histopathological, and ecological studies (Llewellyn and Owen 1960; Paling 1965; Owen 1970; Valtonen et al. 1990; Gannicott and Tinsley 1997, 1998a, b; Rubio-Godoy and Tinsley 2002, 2008), the ultrastructure of their alimentary tract and mechanism of digestion remain mostly unknown. The only other documented ultrastructural studies of this parasite are spermiogenesis (Cable and Tinsley 2001) and development of the tegument (El-Naggar et al. 2021).

Using light microscopy, Llewellyn (1963) described the pharynx and intestine of the larvae (oncomiracidia) of several species of both Monopisthocotylea and Polyopisthocotylea. Although monogenean larvae do not feed until they find and attach to a host, the digestive tract of oncomiracidia is assumed to be functional (Whittington et al. 1999). Some larvae can attach to their host immediately after hatching (e.g., the capsalid *Entobdella soleae* van Benenden and Hesse, 1864 (see Kearn 1981) and the gut of others were reported to contain host blood within a day of attaching to the gills of their host (e.g., the microcotylid *Polylabroides multispinosus* Roubal 1981, see Roubal and Diggles 1993). No published data are available on the ultrastructure of the oncomiracidial alimentary tract. However, Rohde (unpublished data, cited in Whittington et al. 1999), using transmission electron microscopy (TEM), revealed the presence of discrete epithelial cells that are joined by septate junctions in the oncomiracidial alimentary tract of the monopisthocotylean *Encotyllabe chironemi* Robinson 1961. The apical surface of these cells forms lamellae, and their cytoplasm is vacuolated and contains many, small, electron-dense granules and some larger dense bodies, possibly lipid (Whittington et al. 1999).

The present study was conducted to describe, for the first time, the gastrodermis organization in the oncomiracidium, subadult, and adult *D. sagittata* using TEM with the aim of following the mechanism of digestion at different stages of worm ontogeny.

## Material and methods

Rainbow trout (*Oncorhynchus mykiss*) infected with *Discocotyle sagittata* were caught at a Government Fish Hatchery in Cornaa on the Isle of Man, UK. Fish were transported to Bristol University and maintained in aquaria according to the methods outlined by Gannicott and Tinsley (1997). Parasite eggs were collected every 24 h by draining the water from aquaria through a 125-µm sieve; the residue was resuspended in dechlorinated water and decanted into 200-ml crystallizing dishes. Using a dissecting microscope, eggs were collected into Petri dishes and incubated for 3–4 weeks at 13 ± 0.5 °C. Recently emerged oncomiracidia were fixed for TEM (see below) or used to infect naive hosts (see Gannicott and Tinsley 1998b).

Infected fish were pithed, each gill arch was transferred quickly to a Petri dish of dechlorinated water and parasites of different developmental stages were separated and fixed individually for TEM. In addition, 9 larval stages (7 newly hatched oncomiracidia and 2 post-larvae retrieved from their host 24 h post-infection) were fixed together with 7 adults and 4 subadults (with one pair of clamps) that were processed immediately after recovery from their hosts. All specimens were fixed at 4 °C in 2.5% glutaraldehyde buffered with 0.1 M sodium cacodylate, washed overnight in the same buffer, post-fixed for 1 h in cacodylate buffered 1% osmium tetroxide and washed again in buffer, dehydrated in ethyl alcohol, and embedded in Araldite resin. Ultrathin sections were double-stained with uranyl acetate and lead citrate and viewed on a JEOL 1200 EX or 1210 electron microscope operated at 80 kV.

## Ethics

The parasites used in this study were obtained from fish that had naturally acquired their infections and been killed using a Schedule 1 method; therefore, no Home Office licensed procedures were conducted. Nevertheless, ethical considerations followed University of Bristol guidance on animal welfare, and all associated work was covered by a UK Home Office Project Licence.

## Results

### Oncomiracidium

**Fig. 1:**
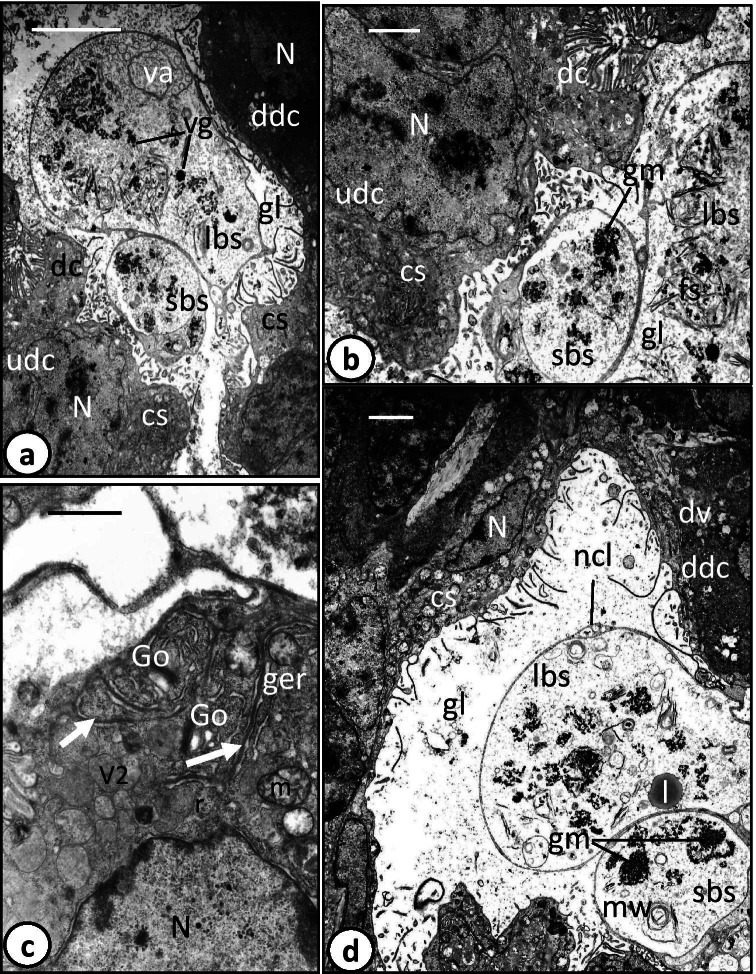
Transmission electron microscope (TEM) photomicrographs of oncomiracidium gastrodermis of *Discocotyle sagittata.* **a** Gastrodermis consisting of digestive cells (dc) and a connecting syncytium (cs). Undifferentiated (udc) and developing differentiated (ddc) cells with large nuclei (N) are present underneath the connecting syncytium (cs). Note large (lbs) and small (sbs) balloon-like structures in the gut lumen (gl) enclosing luminal contents like large, membrane-bound vacuoles (va) with small vesicles and vitelline-like globules (vg). Scale bar = 5 µm. **b** Gastrodermis containing undifferentiated cell (udc) with large nucleus (N) and narrow cytoplasmic layer. Large (lbs) and small (sbs) balloon-like structures contain granular, electron-dense masses (gm) and fibrils-like structures (fs). cs, connecting syncytium; dc, digestive cell; gl, gut lumen. Scale bar = 2 µm. **c** Digestive cell with large nucleus (N) and cytoplasm containing mitochondria (m), Golgi bodies (Go), granular endoplasmic reticulum (ger), ribosomes (r), invaginations of basement membrane (arrow), and digestive vesicles (V2) with moderately electron-dense matrix. Scale bar = 1 µm. **d** Gastrodermis with developing digestive cell (ddc) containing digestive vesicles (dv), and gut lumen (gl) with large (lbs) and small (sbs) balloon-like structures containing lipid-like droplets(l), membranous whorls (mw), and granular, electron-dense masses (gm). Note that each balloon is surrounded by a narrow cytoplasmic layer (ncl). cs, connecting syncytium; N, elongated nucleus. Scale bar = 2 µm

**Fig. 2:**
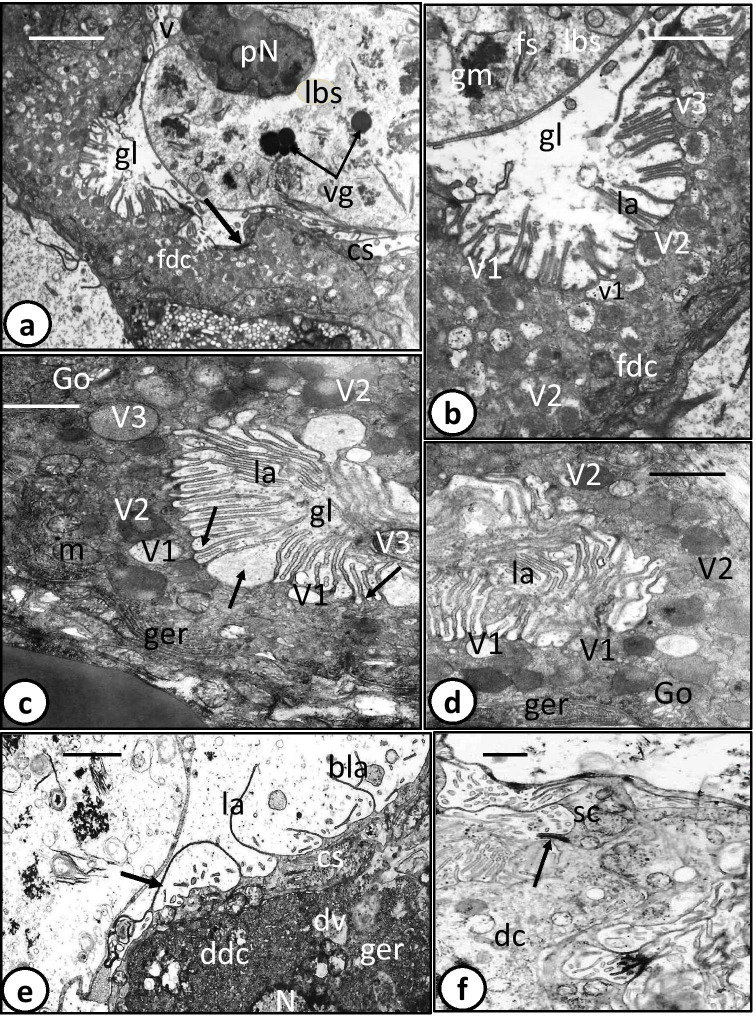
Transmission electron microscope (TEM) photomicrographs of oncomiracidium gastrodermis of *Discocotyle sagittata.* **a** Fully developed digestive cell (fdc) joined to connecting syncytium (cs) via a tight junction (arrow). Note the large balloon-like structure (lbs) with a thin cytoplasmic layer containing pyknotic nucleus (pN) and few vesicles (v) and enclosing vitelline-like globules (vg). gl, gut lumen. Scale bar = 4 µm. **b** Cytoplasm of fully developed digestive cell (fdc) containing pinocytotic vesicles (V1), vesicles with moderately electron-dense matrix (V2) and large vacuole (V3) with small vesicles. fs, fibril-like structures; gl, gut lumen; gm, granular, electron-dense masses; la, luminal lamella of digestive cell; lbs, large balloon-like structure. Scale bar = 2 µm. **c** Cytoplasm of fully developed digestive cell containing Golgi bodies (Go), granular endoplasmic reticulum (ger), mitochondria (m), V1 and V2 digestive vesicles, and V3 vacuoles. Note large and small pits (arrows) between bases of luminal lamella(la), moderately electron-dense core of V2 vesicles and V3 vacuoles in gut lumen (gl). Scale bar = 1 µm. **d** Part of fully developed digestive cell with large and small pinocytotic vesicles (V1), highly electron-dense vesicles (V2), Golgi bodies (Go), granular endoplasmic reticulum (ger), and luminal lamella (la). Scale bar = 1 µm. **e** Connecting syncytium (cs) with unbranched lamella (la), branched lamella (bla), and lamella that re-join the apical surface (arrow). Note the developing digestive cell (ddc) underneath the connecting syncytium (cs). dv, digestive vesicle; ger, granular endoplasmic reticulum. Scale bar = 2 µm. **f** Digestive cell (dc) and connecting syncytium (cs) are joined via a tight junction (arrow). Scale bar = 1 µm

The digestive tract of the oncomiracidium of *Discocotyle sagittata* consists of a subterminal mouth, which leads via a prepharynx into an oval pharynx that opens posteriorly into a large sac-like intestine (gut) with a few lateral diverticula. The intestinal lining consists of digestive cells that are interspersed with a connecting syncytium (Fig. 1a, b). Digestive cells were present in three developmental stages: undifferentiated cells (Fig. 1a, b), developing differentiated cells (Fig. 1d) and fully formed differentiated cells (Fig. 2a–d). The two early stages of digestive cells are often found underneath and close to the connecting syncytium, each possessing a large, centrally located, nucleus (Fig. 1a, b, d). However, the cytoplasm of the undifferentiated cells forms a thin, moderately electron-dense layer around the nucleus and contains few organelles (Fig. 1a, b), while that of the developing differentiated cells is considerably thicker, denser, and appears to contain dense granular endoplasmic reticulum and few incompletely formed digestive vesicles (Figs. 1d and 2e). The digestive cells rest on a basement membrane, invaginations of which extend up to 4.5 µm into the granular cytoplasm (Fig. 1c). The cytoplasm of fully differentiated digestive cells is moderately electron-dense with heterogeneous ground substance (Fig. 1c). The nucleus, with its highly electron-dense perinuclear heterochromatin, is often located at the basal region of the cell surrounded by Golgi bodies, mitochondria, granular endoplasmic reticulum, and ribosomes (Figs. 1c and 2c, d). The apical surface of the fully formed digestive cells bears numerous lamellae, up to 2.5 µm long, with a constant thickness of 0.1 µm (Fig. 2b, c). They often run parallel to each other, perpendicular to the cell surface, and at their bases, the outer plasma membrane evaginates to form pockets (pits), mostly small but sometimes large, in which luminal contents are trapped and later separate to form small (0.1–0.2 µm) or large (0.5–0.7 µm) V1 pinocytotic vesicles (Fig. 2b–d). The latter appear to undergo digestive activities and form membrane-bound V2 vesicles with a moderately electron-dense matrix and dense particles embedded in an electron-lucent ground substance (0.5–1.0 µm) (Fig. 2b–d). Some V2 vesicles have a moderately electron-dense core embedded in more highly electron-dense material (Fig. 2c). Few larger V3 vacuoles (1.0–1.4 µm) with small vesicles can be seen in the apical region of the cell and in the gut lumen (Fig. 2b, c).

**Fig. 3:**
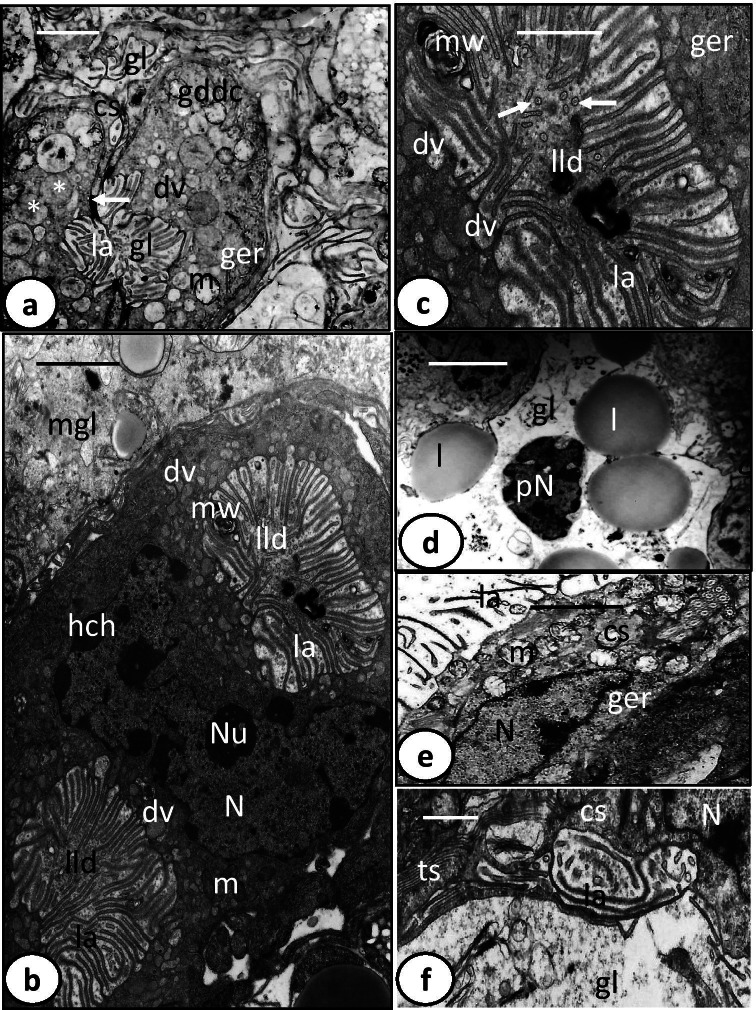
Transmission electron microscope (TEM) photomicrographs of oncomiracidium gastrodermis of *Discocotyle sagittata.* **a** Graduating developing digestive cell (gddc) with narrow gut lumen (gl) and few short lamellae (la). Note the tight junction (arrow) between connecting syncytium (cs) and digestive cell and some large digestive vesicles (*) joining each other. dv, digestive vesicles; ger, granular endoplasmic reticulum; m, mitochondria. Scale bar = 2 µm. **b** Large gradually developing digestive cell with a single large, elongated, irregularly shaped nucleus (N) and two lateral concave lamellated depressions (lld). dv, digestive vesicle; hch, heterochromatin; la, luminal lamella; m, mitochondria; mw, membranous whorls; Nu, nucleolus. Scale bar = 2 µm. **c** Magnified part of concave lateral lamellated depression (lld) containing luminal lamella (la) with central core (arrows), and membranous whorls (mw). dv, digestive vesicles; ger, granular endoplasmic reticulum. Scale bar = 1 µm. **d** Gut lumen (gl) with lipid-like droplets (l), and pyknotic nucleus (pN). Scale bar = 2 µm. **e** Magnified part of connecting syncytium (cs) containing elongated nucleus (N), numerous mitochondria (m), and granular endoplasmic reticulum (ger). la, luminal lamella. Scale bar = 2 µm. **f** Connecting syncytium (cs) with tubular structures (ts), nucleus (N), and interdigitating lamella (la). gl, gut lumen. Scale bar = 1 µm

In some sections, comparatively small developing digestive cells are visible in the area close to the gastrodermis (Fig. 3a). These cells are characterized by a comparatively narrow lumen and considerably shorter luminal lamellae. Their cytoplasm contains numerous mitochondria with short cristae and granular endoplasmic reticulum, both of which are concentrated at the peripheral cytoplasm (Fig. 3a). Moreover, membrane-bound digestive vesicles with different sizes are present but most are moderately electron-dense, and completely formed digestive vesicles with highly electron-dense material are absent (Fig. 3a). Some large vesicles inside these cells appeared to have coalesced and their matrix is continuous (Fig. 3a). Similar larger, more conspicuous developing digestive cells are present just lateral to the main branch of the gut. Each cell has a single large, elongated, irregularly shaped nucleus and two lateral concave lamellated depressions, which likely represent lateral diverticula (Fig. 3b). The nucleus contains a single nucleolus and numerous heterochromatin masses that are highly electron-dense and distributed at the periphery of the nucleus under the nuclear membrane. The cytoplasm of these cells is full of membrane-bound digestive vesicles with moderately electron-dense material (Fig. 3b, c). The luminal lamellae are comparatively long, with some possessing a central electron-dense core in transverse sections (Fig. 3c). The lumen is still narrow but contains few contents as highly electron-dense membranous whorls and lipid-like droplets (Fig. 3b, c).

The digestive cells are connected by apical tight junctions to the connecting syncytium, which covers the lateral and sometimes part of the apical surface of the digestive cells particularly those found in the main branches of the gut (Figs. 2f and 3a). Each nucleus of the connecting syncytium is elongated and lies at the central region of the syncytium surrounded by numerous mitochondria, granular endoplasmic reticulum, ribosomes, a few Golgi complexes, and groups of tubular structures (Fig. 3e, f). The apical surface of the syncytium extends into tubular lamellae (up to 3 µm long), which are more electron-dense and less numerous than those of the digestive cells (Figs. 2e and 3e). Unlike those of the digestive cells, many lamellae are branched, while others recurve and re-join the apical surface (Fig. 2e).

A feature of the oncomiracidium gut is the presence of small and large balloon-like structures consisting of a peripheral narrow layer of cytoplasm that encloses numerous luminal contents including lipid-like droplets, vitelline-like globules, whorls of membranous structures, large membrane-bound vacuoles with probably digested food contents, fine electron-dense fibril-like structures, and fibrous and granulated highly electron-dense material (Figs.1a, b, d and 2a, b, e). Moreover, in some balloons, a pyknotic nucleus with electron-dense particles and disintegrating chromatin patches is seen at the periphery surrounded by a thin layer of cytoplasm containing small vesicles and a few organelles (Fig. 2a). In addition to the previously described contents, the gut lumen contains a single disintegrating pyknotic nucleus surrounded by relatively larger spheroid lipid-like bodies (Fig. 3d). Intact erythrocytes, degenerating digestive cells, hemolyzed hemoglobin, and hematin material were not observed in the gut lumen. Also, hematin granules were not detected in the digestive cells, connecting syncytium or in the gut lumen of the oncomiracidia.

### Adult

**Fig. 4:**
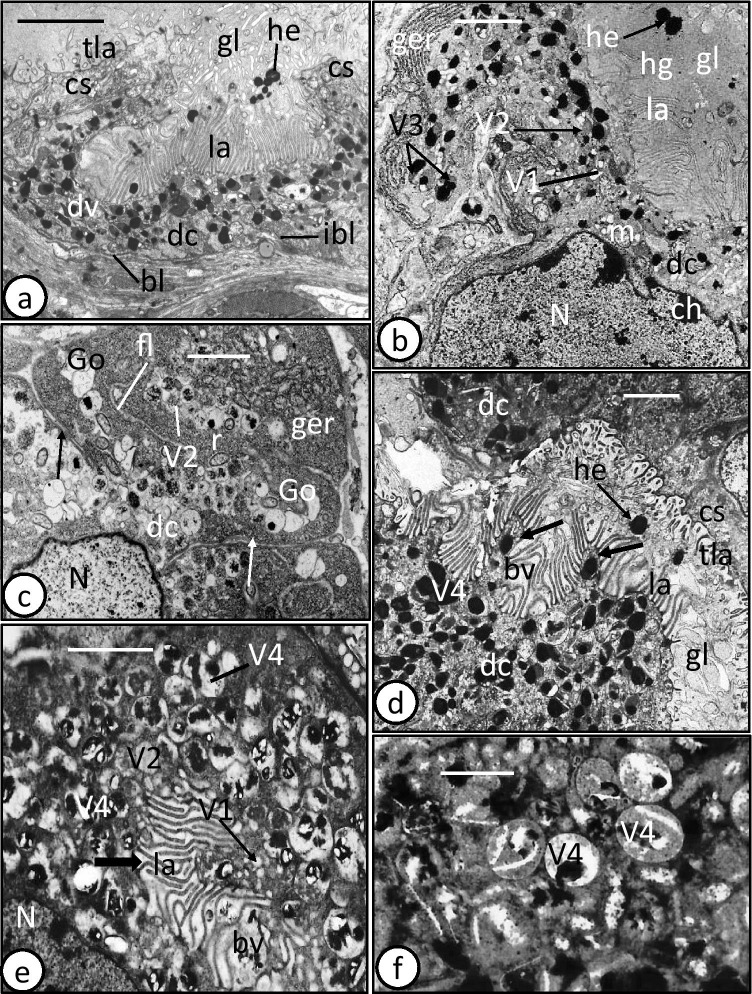
Transmission electron microscope (TEM) photomicrographs of adult and subadult gastrodermis of *Discocotyle sagittata*. **a** Digestive cell (dc) of adult resting on a basal lamina (bl) with many infoldings (ibl) and containing digestive vesicles (dv) at different stages of development. cs, connecting syncytium; gl, gut lumen; he, hematin granules; la, lamella; tla, thin lamella. Scale bar = 4 µm. **b** Digestive cell (dc) cytoplasm of adult with nucleus (N), pinocytotic vesicles (V1), vesicles with moderately electron-dense material (V2), vesicles with moderately and highly electron-dense material (V3), granular endoplasmic reticulum (ger), and mitochondria (m). ch, chromatin; gl, gut lumen; he, hematein granules; hg, hemoglobin; la, lamella. Scale bar = 2 µm. **c** Digestive cell (dc) of adult with long basal lamina infoldings (arrows) enclosing a thin fibrous layer (fl). The cell cytoplasm contains elongated nucleus (N), vesicles with moderately electron-dense particles (V2), Golgi bodies (Go), granular endoplasmic reticulum (ger), and ribosomes (r). Scale bar = 2 µm. **d** Digestive cell (dc) of adult filled with large vacuoles (V4) containing highly electron-dense matrix (hematin). Note the conical outgrowths (arrows) covered with parallel lamellae (la), some of which form small balloon-like vacuole (bv). cs, connecting syncytium; gl, gut lumen; he, hematein granules; tla, thin lamellae. Scale bar = 2 µm. **e** Digestive cell of adult with coated pits (arrow), pinocytotic vesicles (V1), vesicles (V2) with moderately electron-dense material and large vacuoles (V4) with highly electron-dense hematin granules. bv, balloon-like vacuole; la, lamella; N, nucleus. Scale bar = 2 µm. **f** Enlarged portion of the digestive cell cytoplasm showing digestive vacuoles (V4) containing highly electron-dense hematin granules embedded in moderately electron-dense matrix. Scale bar = 1 µm

**Fig. 5:**
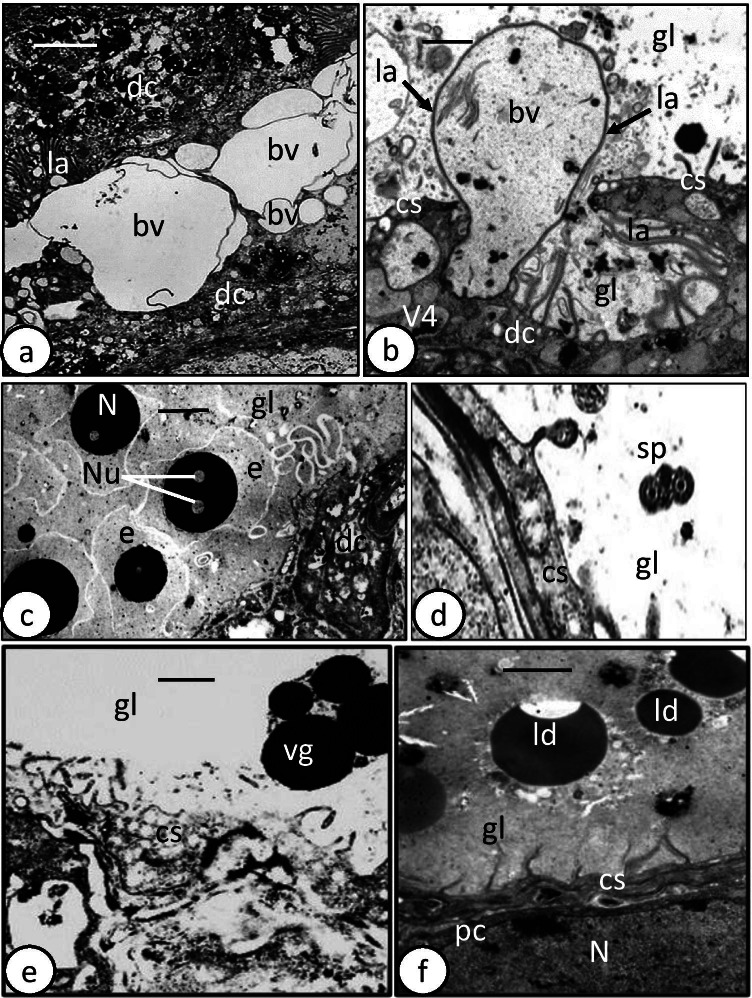
Transmission electron microscope (TEM) photomicrographs of adult and subadult gastrodermis of *Discocotyle sagittata*. **a** Digestive cells (dc) of adult with their lamellae (la), from both sides, protruding to form many small and large balloon-like vacuoles (bv). Scale bar = 2 µm. **b** Large balloon-like vacuole (bv) in adult gastrodermis, enveloped by luminal lamellae (la) of the digestive cell (dc) and enclosing some luminal contents. cs, connecting syncytium; gl, gut lumen; V4, large vacuole containing hematin granules. Scale bar = 1 µm. **c** Gut lumen (gl) of adult containing intact erythrocytes (e), each possessing highly electron-dense nucleus (N) with one or two nucleoli (Nu). dc, digestive cell. Scale bar = 2 µm. **d** Gut lumen (gl) of adult with mature spermatozoa (sp). cs, connecting syncytium. Scale bar = µm. **e** Gut lumen (gl) of adult with vitelline-like globules (vg). cs, connecting syncytium. Scale bar = 1 µm. **f** Gut lumen (gl) of adult with large and small lipid-like droplets (ld). Note the primordial cell (pc) beneath the connecting syncytium (cs). N, nucleus. Scale bar = 2 µm

The intestine of adult *D. sagittata* is bifurcated and possesses numerous median and lateral diverticula. The basic ultrastructure of its gastrodermis is similar to that of the oncomiracidia, but with some additional features. The gastrodermis consists of cup-shaped digestive cells and a connecting syncytium (Fig. 4a, b, d). These digestive cells vary in size and content according to the condition of their digestive activity. In many sections, the apical cytoplasm of these cells protrudes into the gut lumen as conical outgrowths (Fig. 4d). The apical surface also extends into numerous, apparently parallel lamellae, up to 2.7 µm long, with a constant thickness of 0.1 µm (Fig. 4a, d). These lamellae are often separated at their base by coated pits (channels) formed by an invagination of the apical plasma membrane (Fig. 4b, d, e). In some sections of the digestive cells, the apical lamellae extend into the gut lumen and form small and large balloon-like vacuoles enclosing luminal contents (Figs. 4d and 5a, b). The apical portion of the cell contains small pinocytotic vesicles (V1) with homogeneous lightly electron-dense material, while the other part of the cell cytoplasm is filled with larger membrane-bound digestive vesicles with different phases of digestion (Fig. 4a–f). These include vesicles (V2) with moderately electron-dense particles (hemoglobin?) (Fig. 4a, c), vesicles (V3) with moderately and highly electron-dense material (hemoglobin and hematin material) (Fig. 4b, d), and vacuoles (V4) containing highly electron-dense putative hematin granules (crystals) embedded either in electron-lucent or in moderately, electron-dense ground substance (Fig. 4e, f). The digestive cell rests on a thin layer of basal lamina which appears to be separated from the neighbouring cells by a narrow layer of fibrous material (Fig. 4c). Many infoldings of the basal lamina and associated narrow fibrous layer extend into the cytoplasm (Fig. 4c). The nucleus is elongated and lies at the base of the cell (Fig. 4b, c, e). It contains highly electron-dense chromatin patches, particularly beneath the nuclear membrane (Fig. 4b). Granular endoplasmic reticulum, ribosomes, Golgi complexes, and a few small mitochondria are restricted to the cell periphery (Fig. 4b, c).

The gut lumen is mostly filled with whole and intact blood cells, particularly erythrocytes that are packed together and bounded by a plasma membrane with an irregular outline (Fig. 5c). Their cytoplasm is filled with homogeneous, moderately, electron-dense material in which are embedded denser and larger electron-dense particles. Each erythrocyte has a conspicuous, highly electron-dense, nucleus with one or two small nucleoli that are less electron-dense (Fig. 5c). Also, the gut lumen contains products of digestion (hematin granules, Fig. 4a, b, d), hemoglobin of hemolyzed erythrocytes (Fig. 4b), immature and mature spermatozoa (Fig. 5d), vitelline granules (Fig. 5e), and lipid-like droplets (Fig. 5f).

**Fig. 6:**
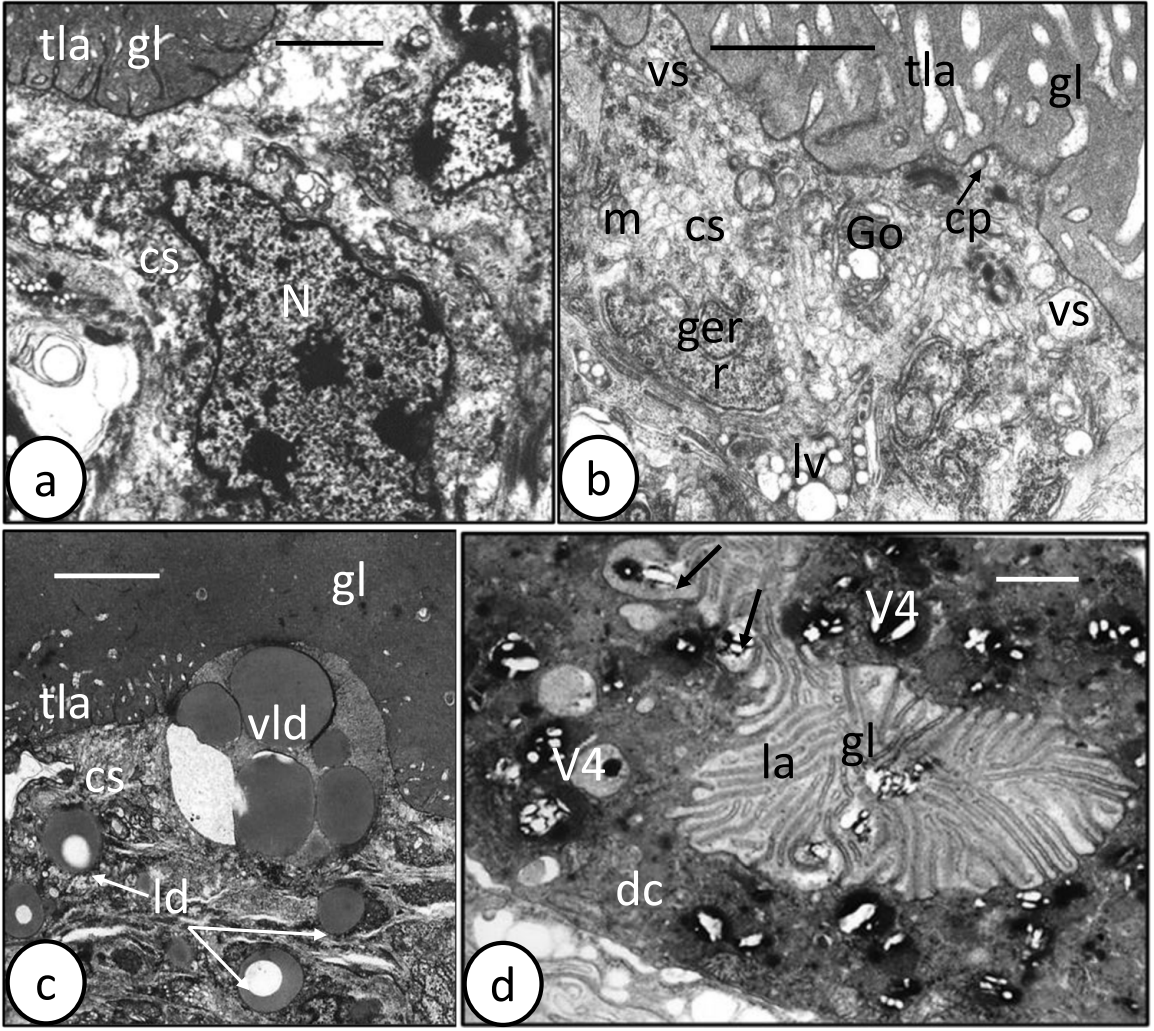
Transmission electron microscope (TEM) photomicrographs of adult and subadult gastrodermis of *Discocotyle sagittata*. **a** Connecting syncytium (cs) of adult with basal, large, elongated nucleus (N) and apical cytoplasm protruding as short thin lamellae (tla) into the gut lumen (gl). Scale bar = 2 µm. **b** Connecting syncytium (cs) of adult with coated luminal pits (cp), small vesicles (vs), large vacuoles containing small vesicles (lv), Golgi bodies (Go), granular endoplasmic reticulum (ger), and mitochondria (m). The apical surface of the syncytium extends into thin lamellae (tla), which expand towards their tips. gl, gut lumen. Scale bar = 1 µm. **c** Connecting syncytium (cs) of adult with isolated lipid-like droplets (ld) and apical, large vacuole packed with lipid droplets (vld). gl, gut lumen; tla, thin lamellae. Scale bar = 2 µm. **d** Digestive cell (dc) of subadult containing various stages of digestive vesicles and large vacuoles (V4) with hematin granules. Note that the bounding membrane of V4 vacuoles fuses with the luminal membrane (arrows) and releases its contents (hematin) into the gut lumen (gl). la, luminal lamellae. Scale bar = 1 µm

In contrast to that of the oncomiracidium, the connecting syncytium in adult *D. sagittata* appears to be metabolically active and is often similar in thickness to the actual digestive cells (Fig. 6a–c). Relatively small vesicles, some aggregated in membrane-bound vacuoles, are scattered throughout the syncytium cytoplasm together with mitochondria, Golgi bodies, lipid-like droplets, and granular endoplasmic reticulum (Fig. 6a–c). The apical surface of the syncytium extends into long thin lamellae (up to 2 µm long), which often expand towards their tip, unlike the uniform digestive cell lamellae (Fig. 6a–d). Coated pits and large vacuoles containing a mass of packed lipid droplets are also present at the apex of the syncytium (Fig. 6b, c). No hematin granules are apparent in the connecting syncytium. Prominent nuclei tend to lie in folds of the gastrodermis (Fig. 6a). Putative primordial cells were observed underlying both the connecting syncytium and digestive cells (Fig. 5f). In subadults (with one pair of clamps) and post-larvae, retrieved from their host 24 h post-infection, the digestive cells closely resemble those of the adult in having various stages of digestive vesicles (V1–V3) and large vacuoles (V4) (Fig. 6d). The latter (V4), which contain hematin granules, appear to fuse with the luminal membrane and extrude their contents into the gut lumen (Fig. 6d).

## Discussion

Apart from the unpublished study by Rohde (cited in Whittington et al. 1999) on the oncomiracidium of the monopisthocotylean *E. chironemi*, this is the first ultrastructural study of the oncomiracidium gastrodermis in the Monogenea. The gastrodermis of *D. sagittata* follows the same cellular organization reported in other previously described blood-feeding polyopisthocotyleans (e.g., Rohde 1973; Halton 1975; Halton et al. 1968; Bogitsh 1993; Poddubnaya et al. 2015), being composed of digestive cells alternating with a connecting syncytium. However, the present study revealed some new features that can be used to distinguish between the gastrodermis of the oncomiracidium and that of both adult and subadult worms. An interesting feature of the oncomiracidium gastrodermis is that the digestive cells are present in three developmental forms: undifferentiated, developing differentiated, and differentiated (presumably functioning) cells, whereas the adult digestive cells are mainly found in a single functioning state with variable size and contents, although primordial cells were observed underneath both digestive cells and connecting syncytium. Also, the apical cytoplasm of the adult digestive cells forms conical outgrowths, a feature which is absent in the oncomiracidium. In contrast to that of the oncomiracidium, the connecting syncytium in adult and subadults is thought to be metabolically active since it contains relatively small vesicles, some of which are aggregated in membrane-bound vacuoles, as well as large vacuoles filled with compact lipid droplets. Moreover, the thin lamellae of the adult are more numerous and larger, and their terminal portions are expanded if compared with those of the oncomiracidium. Parallel, tubular, membranous structures are characteristic of the apical cytoplasm of the connecting syncytium of the oncomiracidium.

The presence of the oncomiracidium digestive cells in three developmental forms reflects the need of the larval gastrodermis to increase in size to form the new diverticula of the maturing parasite, and preparing the larva for blood feeding, which appears to start soon after the oncomiracidium attaches to the host’s gill tissues. Poddubnaya et al. (2015) detected similar undifferentiated and developing differentiated digestive cells in the adult polyopisthocotylean *Chimaericola leptogaster* Leuckart 1830 and suggested a permanent renovation of these cells in chimaercolids. The finding of large digestive cells with apparently dividing nucleus and two lateral lamellated depressions in the oncomiracidium gastrodermis of *D. sagittata* supports the idea of cell turnover, with division of undifferentiated cells to form differentiated cells with lamellae that increase in length with increasing cell size. This process may lead to the formation of lateral diverticula. The luminal surface of the connecting syncytium of the oncomiracidium gastrodermis always contains numerous groups of parallel tubular structures, the functions of which are unknown, but possibly serve as a membranous stock for the development of surface lamellae.

The gut lumen of the free swimming oncomiracidium is free of intact host erythrocytes or hematin granules, and its connecting syncytium has no evidence of metabolic activity, features which are apparent in adult and subadult *D. sagittata*. Moreover, the gut lumen of the oncomiracidium contains comparatively large balloon-like, nucleated cytoplasmic structures, enclosing luminal contents in the form of lipid-like droplets, large and small vacuoles, vitelline-like globules, and other nutritional material. Luminal balloons were observed in adults, but they are smaller, their cytoplasmic layer has no nuclei, and they enclose limited luminal contents. The origin of these structures in the oncomiracidium is unknown, but it seems most likely that they are formed from protrusions of the apical part of the connecting syncytium since their cytoplasmic roots are very close to and interdigitate with the outer lamellated surface of the connecting syncytium. Moreover, the enveloping cytoplasmic layer of the balloon contains the same cytoplasmic contents of the connecting syncytium. This balloon-like structure might serve as a net that captures and accumulates the luminal contents bringing them closer to the surface lamellae of the digestive cells, which in turn traps food material into the pinocytotic vesicles of the outer plasma membrane. Structures termed “lamellate bubbles” were reported in adult cecal epithelium of the polyopisthocotylean *C. leptogaster* (see Poddubnaya et al. 2015). However, these lamellated bubbles are formed over the apical surface of the digestive cells and some free bubbles containing residual digested bodies were observed in the cecal lumen. Poddubnaya et al. (2015) suggested that the residual bodies and digestive cell material surrounded by the bubbles are discharged into the cecal lumen and considered this as an alternative way of digestive product elimination in chimaericolids.

Variations in the ultrastructural organization of the gastrodermis are reported among different families of the Polyopisthocotyla (Halton et al. 1968; Brennan and Ramasamy 1996; Santos et al. 1998; Poddubnaya et al. 2015). As in the present study, and in the diclidophorid *Diclidophora merlangi* (Kuhn, in Nordmann, 1832) and the chimaericolid *C. leptogaster*, the digestive cells alternate with the connecting syncytium within the cecal wall (Halton et al. 1968; Poddubnaya et al. 2015), whereas in the thoracotylid *Pricea multae* Chauhan 1945 and hexabothriids *Rajanchocotyle emarginata* (Olsson 1876) Sproston 1946 and *Callorhynchocotyle callorhynchi* (Manter 1955) Boeger, Kritsky and Pereira 1989, the digestive cells occur in groups and may project deep into the lumen (Brennan and Ramasamy 1996; Poddubnaya et al. 2015). Primordial (undifferentiated) cells were frequently observed underneath the digestive cells and connecting syncytium of adult *D. sagittata.* In this respect, the gastrodermis of adult *D. sagittata* resembles that of other previously studied polystomatids, thoracocotylids, and hexabothriids (Tinsley 1973; Brennan and Ramasamy 1996; Poddubnaya et al. 2015, respectively). It was suggested that such a phenomenon could form a permanent process of renovation of these cells in studied polyopisthocotyleans (Poddubnaya et al. 2015). The connecting syncytium of the gastrodermis is smooth with no lamellae in the non-blood-feeding polystomatids of the genera *Polystomoides*, *Polystomoidella*, *Neopolystoma*, and *Concinnocotyla* from chelonians and a lungfish (Rohde 1973; Allen and Tinsley 1989; Watson and Whittington 1996), and in the blood-feeding genus *Protopolystoma* from *Xenopus* (see Tinsley 1973). The digestive cells and connecting syncytium in both the oncomiracidium and adult *D. sagittata* are connected at the luminal surface, by septate junctions. Similar junctions were reported in other polyopisthocotylean monogeneans, e.g., *D. merlangi* (see Halton et al. 1968), *Polystomoides*, *Neopolystoma*, and *Concinnocotyla* (see Rohde 1973; Watson and Whittington 1996) and in the chimaericolid *C. leptogaster* and hexabothriids *R. emarginata* and *C. callorhynchi* (see Poddubnaya et al. 2015), but neither in the thoracocotylid *P. multae* (see Brennan and Ramasamy 1996) nor in the microcotylid *Atriaster heterodus* Lebedev & Parukhin 1969 (see Santos et al. 1998). The mechanism of digestion in the oncomiracidium gastrodermis resembles that reported in other tissue feeding monopisthocotyleans (see for example, Arafa et al. 2013).

In the oncomiracidium of *D. sagittata*, the small and large pinocytotic vesicles (V1) in the outer part of the cell can withdraw nutrients from the gut lumen and subsequently pass-through intracellular digestion by enzymes produced by GER-Golgi complexes resulting in the accumulation of digested material within large membrane-bound vesicles (V2). The latter vesicles come into close contact with the outer plasma membrane, and their residual contents of digestion may be eliminated by exocytotic mechanism through the outer plasma membrane.

The digestive cells in subadult and adult *D. sagittata* resemble those described in other polyopisthocotyleans, in that they contain digestive vesicles at various stages of development (Halton 1975; Bogitsh 1993; Poddubnaya et al. 2015), namely pinocytotic vesicles (V1), vesicles (V2) with moderately electron-dense particles (hemoglobin?), vesicles (V3) with moderately and highly electron-dense material (hemoglobin and hematin-like material), and vacuoles (V4) containing highly electron-dense putative hematin granules (crystals) embedded either in electron-lucent or in moderately, electron-dense ground substance. Exocytotic vacuoles (V4) with hematin granules were also seen at the apical surface of these cells. Therefore, the mechanism of digestion in subadult and adult *D. sagittata* might resemble that described for other blood-feeding polyopisthocotyleans. According to Halton (1975, 1997), the digestive cells of blood-feeding monogeneans function in the uptake and intracellular digestion of host-blood hemoglobin. Golgi complex and associated organelles, particularly the endoplasmic reticulum, are involved in the synthesis of digestive enzymes suggesting that the endocytosis of host macromolecules occurs. These small pinocytotic vesicles are responsible for the transmission of absorbed hemoglobin for intracellular digestion by enzymes of GER-Golgi origin (Halton 1975).

In polyopisthocotyleans, both apocrine and holocrine processes are involved in eliminating the accumulated waste hematin from digestive cells; in adult and subadult *D. sagittata*, this process seems apocrine-based. Holocrine and/or apocrine processes were suggested for polystomatids *Polystomoides malayi* Rohde 1963 and *P. renschi* Rohde 1965 by Rohde (1973), unidentified species of *Polystomoides* by Allen and Tinsley (1989), the thoracocotylid *P. multae* by Brennan and Ramasamy (1996), polystomatid *Neopolystoma spratti* Pichelin 1995 by Watson and Whittington (1996), and the hexabothriids *R. emarginata* and *C. callorhynchi* by Poddubnaya et al. (2015), while apocrine process was reported for the chimaericolid *C. leptogaster* (see Poddubnaya et al. 2015) and the monopisthocotylean *Euzetrema knopffleri* Combes 1965, which has a partly hematophagous diet (Fournier 1978).

The gastrodermis lumen of adult *D. sagittata* contained digestive products mixed with vitelline globules, spermatozoa, and lipid-like droplets. Similar components were reported in other polyopisthocotyleans, e.g., *C. callorhynchi* by Poddubnaya et al. (2015) who suggested that these reproductive materials are released into the cecal lumen via the genito-intestinal canal, which is generally considered to be an outlet for expended reproductive material (Smyth and Halton 1983). Indeed, in the polystomatid monogeneans *Pseudodiplorchis americanus* (Rodgers & Kuntz 1940) Yamaguti 1963 and *Neodiplorchis scaphiopodis* Rogers 1941, it seems nutrients from in utero larva can be recycled to the adult digestive system if the annual opportunity for transmission is missed (Cable and Tinsley 1991). The apical lamellae of some *D. sagittata* digestive cells extended into the gut lumen as a balloon-like vacuole, enclosing luminal contents. Similar bubble-like structures in the chimaericolid *C. leptogaster* were described as another form of digestive product elimination through which residual bodies and digestive cell material surrounded by the bubble are discharged into the cecal lumen (Poddubnaya et al. 2015). In the hexabothriids *R. emarginata*, homologous bubbles were observed but formed by means of lateral lamellae of the connecting syncytium over the apical lamellated surface of the digestive cells (Poddubnaya et al. 2015). Another interesting feature of *D. sagittata* is that the apical cytoplasm of the adult digestive cells forms conical outgrowths, a feature which is absent in the oncomiracidium. Outgrowths, terminating with long narrow processes, were observed in the luminal surface of the connecting syncytium (not the digestive cells) of *C. leptogaster.* It seems possible that these outgrowths with the associated lamella in *D. sagittata* and associated long processes in *C. leptogaster* may serve to increase the surface area available for absorption of nutrients.

A surprising feature of the gastrodermis of *D. sagittata* is the metabolically active connecting syncytium. Originally, the connecting syncytium of polyopisthocotyleans was ascribed a supportive role (Tinsley 1973; Halton 1976), but as the connecting syncytium of *Pricea multae* contains numerous vesicles, mitochondria, ER, and Golgi bodies, it was suggested it might also be involved in absorption of macromolecules (Brennan and Ramasamy 1996). The connecting syncytium of *D. sagittata*, however, appears much more active than that of any other monogenean described. Other gill blood-feeding polyopisthocotyleans examined have lamellae on both digestive cells and the connecting syncytium, although the lamellae tend to be longer on the digestive cells (Brennan and Ramasamy 1996). The advantages conferred by an increased surface area of the syncytium by the lamellae are presumably related to nutrient uptake. The presence of alkaline phosphatase, which is often associated with membrane ATPases, among the lamellae in the gastrodermis syncytium of *D. merlangi* suggests that this tissue is involved in the uptake of low molecular weight nutrients (Halton et al. 1968). Gap junctions between the syncytium and underlying parenchyma in *D. merlangi* (see Halton et al. 1968), *Pseudodiplorchis americanus*, and *N. scaphiopodis* (cable personal observations) provide further support for a function in nutrient absorption.
